# The Fitness Effects of Random Mutations in Single-Stranded DNA and RNA Bacteriophages

**DOI:** 10.1371/journal.pgen.1000742

**Published:** 2009-11-26

**Authors:** Pilar Domingo-Calap, José M. Cuevas, Rafael Sanjuán

**Affiliations:** 1 Instituto Cavanilles de Biodiversidad y Biología Evolutiva, València, Spain; 2 Departamento de Genética, Universitat de València, València, Spain; University of California Davis, United States of America

## Abstract

Mutational fitness effects can be measured with relatively high accuracy in viruses due to their small genome size, which facilitates full-length sequencing and genetic manipulation. Previous work has shown that animal and plant RNA viruses are very sensitive to mutation. Here, we characterize mutational fitness effects in single-stranded (ss) DNA and ssRNA bacterial viruses. First, we performed a mutation-accumulation experiment in which we subjected three ssDNA (ΦX174, G4, F1) and three ssRNA phages (Qβ, MS2, and SP) to plaque-to-plaque transfers and chemical mutagenesis. Genome sequencing and growth assays indicated that the average fitness effect of the accumulated mutations was similar in the two groups. Second, we used site-directed mutagenesis to obtain 45 clones of ΦX174 and 42 clones of Qβ carrying random single-nucleotide substitutions and assayed them for fitness. In ΦX174, 20% of such mutations were lethal, whereas viable ones reduced fitness by 13% on average. In Qβ, these figures were 29% and 10%, respectively. It seems therefore that high mutational sensitivity is a general property of viruses with small genomes, including those infecting animals, plants, and bacteria. Mutational fitness effects are important for understanding processes of fitness decline, but also of neutral evolution and adaptation. As such, these findings can contribute to explain the evolution of ssDNA and ssRNA viruses.

## Author Summary

The fitness effects of mutations are the raw material for natural selection. It has been shown that point mutations typically have strongly deleterious effects in plant and animal RNA viruses, whereas cellular organisms are comparatively more robust. Here, we characterize the fitness effects of random mutations in DNA viruses and compare them with those found in RNA viruses, using six phage species of similar genome sizes. To achieve this goal, we introduced mutations by chemical and site-directed mutagenesis, identified the genetic changes by sequencing, and quantified their fitness effects using growth-rate assays. In all cases, mutations had a strong average impact on fitness. We conclude that mutational sensitivity is a general property of viruses with small genomes and discuss the evolutionary implications of these findings.

## Introduction

Mutational fitness effects are important for understanding the genetic variability of populations, the relative roles of natural selection and drift, the origin of sex and recombination, or the ability to produce evolutionary innovations, among other processes [1]–[4]. Further, they are of practical relevance to several fields, including complex human disease [5] and conservation genetics [6]. A simple parameter describing mutational fitness effects is the mean selection strength, defined as the average change in fitness caused by random mutations. However, it is also important to determine their variance and the shape of their statistical distribution. A classic approach to achieve these goals is the mutation-accumulation experiment, in which lines derived from a founder clone are propagated at the smallest possible population size to minimize selection, thereby allowing mutations to accumulate [7],[8]. A more direct and powerful approach consists of genetically engineering random mutants, although this has been done far less often due to the greater difficulty of the task [9].

Some progress has been made in characterizing mutational fitness effects. For instance, in *Escherichia coli* more than 90% of gene knock-outs are viable [10] and random insertions caused by transposition reduce fitness by 3% on average [11]. In nematodes, the vast majority of nucleotide substitutions have very small fitness effects [12], whereas in humans few amino acid substitutions have effects greater than 10% and about 30% evolve neutrally [9]. However, the distribution of mutational fitness effects is complex and large differences between species may exist [9]. For example, in some RNA viruses random nucleotide substitutions reduce fitness by nearly 50% on average and up to 40% are lethal [13],[14]. This extreme mutational sensitivity contrasts with the greater robustness of cellular organisms. Mutational robustness is thought to emerge from the presence of alternative metabolic pathways, genetic redundancy, or modularity, but these buffering mechanisms are usually not found in RNA viruses due to their compact genomes [15].

Viruses are a unique experimental system for characterizing mutational fitness effects because it is relatively easy to engineer sets of random single-nucleotide substitutions. This has been done previously for single-stranded (ss) RNA viruses, but not for ssDNA viruses. These two types of viruses have similar genome sizes and therefore might be expected to be equally sensitive to mutation. On the other hand, given the obvious ecological [16], evolutionary [17], and genetic [18] differences between ssDNA and ssRNA viruses, differences in robustness would not be surprising. For instance, ssRNA viruses show higher mutation rates [19]–[21], possibly selecting for greater robustness [22]–[24], although this might have also promoted genome compression [25]–[27]. Second, replicase genes represent a large portion of the genome of RNA viruses but are not encoded by ssDNA viruses, which use host DNA polymerases. Since replicase genes are typically highly constrained, this might also lead to differences in robustness between ssRNA and ssDNA viruses. Finally, some ssDNA viruses encode two scaffolding proteins, apparently introducing some redundancy in the process of capsid assembly [28].

**Figure 1:**
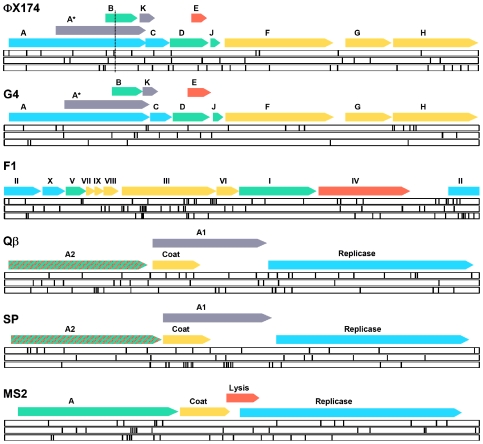
Genome structure of phages ΦX174, G4, F1, Qβ, SP, and MS2, and location of the genetic changes fixed in mutation-accumulation lines. Genome sizes are 5.4 kb, 5.6 kb, 6.4 kb, 4.2 kb, 4.3 kb, and 3.6 kb, respectively. Protein-coding regions represent between 91% (F1) and 96% (ΦX174) of the total genome, respectively. Arrows indicate the location of each cistron and colors represent broad functional categories (blue: replication; green: particle assembly; yellow: coat; red: lysis/extrusion; grey: other/unknown). However, several genes are multifunctional; for instance A2 is involved in phage assembly and lysis. The location of each of the mutations fixed in the mutation-accumulation lines is shown within white bars, each bar corresponding to an independent line (see Table S1 for details). The three DNA phages have circular genomes but are shown in linear form for convenience. The genomes of ΦX174 and G4 are shown collinearly to indicate gene homology. However, by convention, the first position of the ΦX174 genome is assigned to the unique Pst I cleavage site (vertical dashed line).

Here, we compare mutational fitness effects in ssDNA and ssRNA viruses using six phages which can infect the same *E. coli* strain under identical environmental conditions [29]: the microviruses ΦX174 and G4, the inovirus F1 (ssDNA viruses), the alloleviviruses Qβ and SP, and the levivirus MS2 (ssRNA viruses) (Figure 1). First, we carried out a mutation-accumulation experiment in which we subjected phages to plaque-to-plaque passages and chemical mutagenesis, quantified changes in fitness, and sequenced the ancestral and evolved genomes. Second, we used site-directed mutagenesis to determine the mean strength of selection more accurately and to characterize the distribution of fitness effects in ΦX174 and Qβ. We demonstrate that ssDNA and ssRNA phage exhibit very similar mutational fitness effects.

## Results

### Mutation-accumulation

Preliminarily, we adapted each phage to our laboratory conditions by performing serial passages at high population sizes, until all lineages reached stable fitness values. For each phage, three independent mutation-accumulation lineages were initiated by picking single plaques at random from the adapted populations. Phage supernatants from plaques were subjected to chemical mutagenesis using nitrous acid and plated to isolate new plaques, a protocol that was continued until plaque sizes became drastically reduced. Under these conditions, selection is minimized and therefore, except for highly deleterious or lethal mutations, random genetic drift and mutational pressure are the only factors driving the fixation of mutations [9]. Growth rates were then assayed and the fitness values relative to the non-mutated genotype were obtained as growth rate ratios.

The change in fitness was homogeneous among species (Table 1, nested ANOVA: *P* = 0.769) and there were no significant differences between the DNA and RNA groups (*P* = 0.605). After sequencing the six ancestral and the 18 evolved genomes (GenBank accession numbers GQ153912-GQ153935) we found that DNA and RNA phages had accumulated 157±3 and 146±1 nucleotide substitutions in total, respectively (Table 1, Figure 1, nested ANOVA: *P* = 0.802). We calculated the per-mutation effect by dividing the fitness loss of each lineage by the number of mutations accumulated and again, this did not reveal significant differences between DNA and RNA phages (*P* = 0.870). We therefore conclude that selection strength is similar in the two groups. F1 appeared to show the highest level of robustness, but differences between species were non-significant (*P* = 0.088) and a Tukey's post-hoc test indicated that the six phages formed a single coherent group.

**Table 1:** Summary of the results obtained in the mutation-accumulation experiment.

Genetic material	Phage^a^	Passage number	Relative fitness	Mutation number	Fitness effect per mutation^b^
ssDNA	ΦX174	60.3±3.0	0.235±0.004	16.0±3.0	−0.051±0.008
	G4	34.0±11.7	0.313±0.103	10.7±2.9	−0.072±0.169
	F1	45.7±9.9	0.342±0.121	25.7±2.8	−0.027±0.007
	Average	46.7±5.9	0.297±0.049	17.4±2.6	−0.050±0.009
ssRNA	Qβ	14.3±3.7	0.295±0.053	18.7±2.4	−0.040±0.008
	SP	7.0±2.1	0.205±0.089	15.0±2.3	−0.057±0.014
	MS2	7.3±0.3	0.313±0.081	15.0±1.0	−0.046±0.003
	Average	9.6±1.7	0.284±0.031	16.2±1.2	−0.047±0.053

a For each phage, the average of three independent lines ± SEM is shown.

b Calculated by subtracting one from the relative fitness and dividing by the number of mutations.

However, the number of passages required to reach similar fitness losses or mutation numbers was higher on average for DNA phages (Table 1, nested ANOVA, *P* = 0.010). This might be due to their lower spontaneous mutation rates or to lower susceptibility to the mutagen. In both groups, the proportion of transitions was high, and this excess was significantly more marked in DNA phages (94.9% versus 84.9%, Fisher's exact test: *P* = 0.004). Since nitrous acid induces mainly transitions, this suggests that, in DNA phages, most substitutions were caused by the mutagen, whereas in RNA phages there was a greater contribution of spontaneous mutation. This could explain why more passages were required in the former group, although differences in susceptibility to the mutagen or in the proportion of transitions among spontaneous mutations cannot be discarded. Importantly, the higher transition/transversion ratio of DNA phages did not result in a significantly higher fraction of synonymous substitutions (nested ANOVA, *P* = 0.484) and therefore is unlikely to have biased the above selection strength estimates.

### Site-directed mutagenesis

We constructed 45 clones of ΦX174 and 42 clones of Qβ carrying single-nucleotide substitutions, choosing the target site and the substitution at random. First, we amplified the viral genomes by PCR using mutagenic primers, transfected *E. coli* with the PCR products, picked single plaques, and verified the presence of the mutation by sequencing. Some transfected cultures failed to form plaques, suggesting that the engineered mutation was lethal for the virus. We first sequenced the region of the mutagenesis PCR product flanking the target site to verify that there were no additional changes. Then, control assays were carried out in which the entire mutagenesis protocol was repeated using PCR primers that did not carry the mutation. By comparing the numbers of plaques formed in mutagenesis and control assays, we showed that 9 and 12 mutations were lethal in ΦX174 and Qβ, respectively (Figure S1), i.e. a lethal fraction of *p_l_* = 0.20 and *p_l_* = 0.29, respectively. These two proportions did not differ significantly (Fisher's exact test, *P* = 0.454). All lethal mutations found in ΦX174 produced amino-acid changes, whereas in the case of Qβ, one was synonymous (U2379A) and one intergenic (G1329A).

We measured the growth rate of viruses carrying viable substitutions to obtain the distribution of mutational fitness effects. Growth rates were also determined for the above control samples and corrected means and variances were obtained by subtracting the mean and variance of the control group from the grand mean and variance of the mutants. In ΦX174, this yielded a mean selection strength of 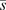 = −0.301 including all (lethal or viable) mutations, with variance *V*(*s*) = 0.162. For viable mutations only, 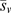 =  −0.126 and *V*(*s_v_*) = 0.047. In Qβ, 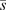 = −0.359, *V*(*s*) = 0.181, 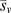 = −0.103 and *V*(*s_v_*) = 0.018 (Table 2). There were no significant differences in mean selection strength between the two phages (nested ANOVA for viable mutations: *P* = 0.633; Mann-Whitney test for all mutations: *P* = 0.336). The above correction using controls implies that our inferences of the mean and variance should be free of experimental error or bias resulting from the presence of additional mutations or changes in the assay conditions, although it is still possible that the fitness values of some individual mutants were altered by the presence of additional mutations.

**Table 2:** Summary of the results obtained in the site-directed mutagenesis experiment^a^.

	ΦX174	Qβ
*p_l_*	0.20	0.29
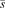	−0.301	−0.359
*V(s)*	0.162	0.181
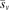	−0.126	−0.103
*V(s_v_)*	0.047	0.018
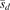	−0.195/−0.176	−0.152/−0.121
*V(s_d_)*	0.031/0.038	0.024/0.033
*p_d_*	0.52/0.57	0.45/0.57
*p_n_*	0.23/0.28	0.14/0.26

a *p_l_, p_d_, p_n_*: fraction of lethal, deleterious, and neutral mutations, respectively. 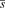: mean selection strength. *V*(*s*): variance of mutational fitness effects. Sub-indexes *v* and *d* refer to all viable mutations and deleterious (non-lethal) mutations, respectively. *p_l_*, 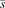, *V*(*s*), 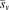, and *V*(*s_v_*) were obtained directly from the data. 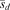 and *V*(*s_d_*) were estimated by non-linear regression from negative *s*-values only and used to infer *p_d_* and *p_n_* (95% confidence intervals are shown in these cases).

**Figure 2:**
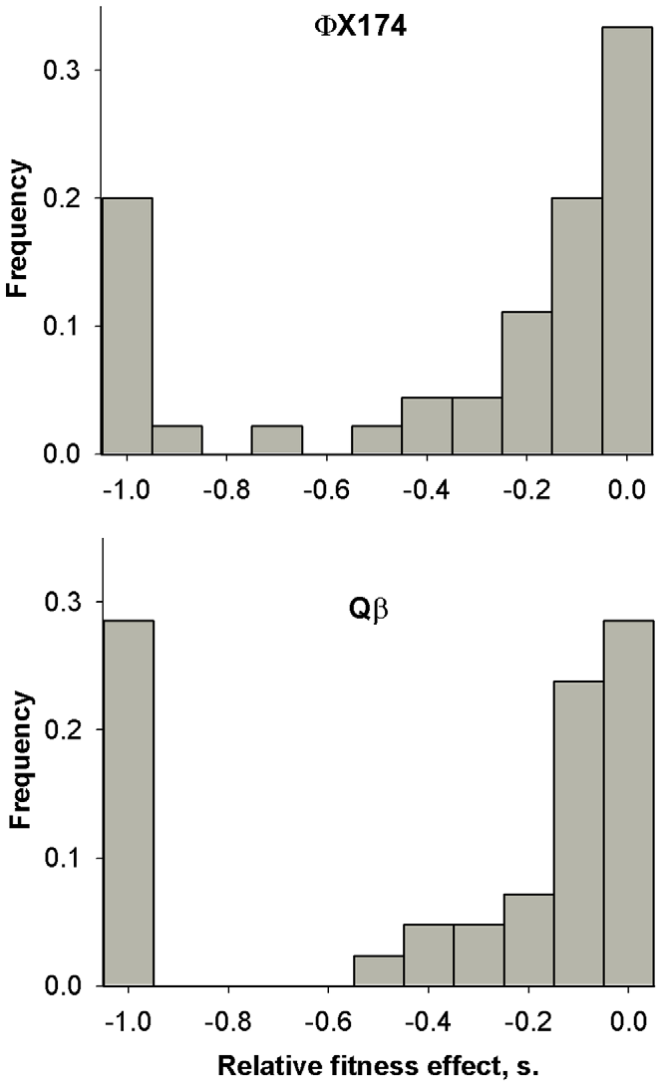
Distribution of fitness effects caused by single-nucleotide substitutions in phages ΦX174 and Qβ. Forty-five and 42 mutations, respectively, were obtained by site-directed mutagenesis and assayed for fitness. The effect of each individual mutation is provided in Table S2 and Table S3.

The distribution of fitness effects of viable mutations was highly skewed, with deleterious substitutions of small effect being more abundant than those of large effect (Figure 2), a property shared by most model systems studied [9]. Focusing on viable mutations with negative *s*-values, we performed non-linear regression to characterize the shape of the distribution. In ΦX174, an exponential model with an expected mean selection strength against deleterious mutations of 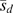 = −0.186±0.005 described the data satisfactorily (*R* ^2^ = 0.989) and was as accurate as a Gamma (*R* ^2^ = 0.989) or a Beta (*R* ^2^ = 0.985) model (partial *F*-test, *P*>0.25 in both cases). Other statistical models were not considered here. In Qβ, the data were better described by the Gamma distribution (*R* ^2^ = 0.975) with an expected 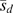 = −0.136±0.008. The Beta model provided a similarly good fit (*R* ^2^ = 0.973), whereas the exponential model was slightly worse (*R* ^2^ = 0.965, *P* = 0.025). Finally, we must emphasize that mutants with positive values were not used for these inferences, which implies that they were assumed to be neutral or beneficial. Neglecting beneficial mutations, the fraction of deleterious to total mutations should thus be *p_d_* =  (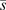
−*p_l_*)/*s_d_* and the fraction of neutral to total mutations simply *p_n_* = 1−*p_l_*−*p_d_* (Table 2). However, classifying mutations as neutral or deleterious is somewhat unnatural since the difference between a deleterious mutation of infinitesimally small effect and a neutral mutation is merely formal.

## Discussion

Previous site-directed mutagenesis studies have shown that most random nucleotide substitutions are strongly deleterious in animal and plant ssRNA viruses. In Vesicular stomatitis virus (VSV), *p_l_* = 0.40, 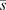 = −0.48 and 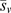 = −0.13 [14], whereas in the unrelated Tobacco etch virus (TEV), *p_l_* = 0.41, 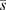 = −0.49, and 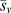 = −0.13 [13]. After performing similar experiments with phage Qβ, we confirm that ssRNA viruses are extremely sensitive to mutation in general. Roughly speaking, the probability that a random single nucleotide substitution is lethal for an ssRNA virus is one third or higher, while viable mutations reduce fitness by 10–13% on average. The observed fraction of lethals was lower in Qβ than in VSV or TEV, which might reflect real biological differences or may be a consequence of methodological issues. Concerning the shape of the distribution, viable mutations of small effect are more abundant that those of large effect. The specific statistical model that better describes the data varies, but the Gamma and the Beta distributions are generally accurate. However, larger mutant collections would be needed to increase the statistical power of inferences about the distribution of mutational fitness effects.

We analyzed ΦX174 using the same methodology and under the same environment as Qβ to make the comparison between ssDNA and ssRNA viruses. The two phages did not significantly differ in the fraction of lethal mutations or the average strength of selection, suggesting that ssDNA and ssRNA viruses are similarly sensitive to mutation. The strong parallel shown by viruses as different as ΦX174 and Qβ probably stems from the fact that both have small genomes with few and short non-coding regions, several multifunctional proteins, and little genetic redundancy [15],[30]. Differences between ΦX174 and Qβ were only minor. For instance, deleterious (non-lethal) effects seemed to follow a simple exponential law in ΦX174 whereas Qβ deviated from this model, probably because there were more mildly deleterious mutations and fewer strongly deleterious ones (Figure 2, Table 2), although the fraction of lethals might be higher in the latter.

The similar robustness shown by ssDNA and ssRNA viruses was also supported by the mutation-accumulation experiment, in which we examined three phage species of each group and found no significant differences in mean selection strength. Differences between species within the two groups were weak or absent, although F1 appeared to be the most robust phage. Related to this, it is worth noting that F1 has fewer overlapping genes. This might be related to the fact that inoviruses have filamentous capsids, which are structurally less constrained than icosahedral ones and can hence accommodate larger genomes [31],[32]. However, further work is needed to elucidate whether F1 really differs from the other phages in terms of robustness.

It is possible to compare directly the mean selection strengths estimated by mutation-accumulation and site-directed mutagenesis, since the same viruses and the same environment were used. Because lethal mutations cannot be sampled during plaque-to-plaque passages we expected the former to be lower. However, even after excluding lethals, the 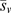
values obtained by site-directed mutagenesis experiments were approximately twofold higher. The most likely explanation for this discrepancy is that, since the population size within plaques necessarily exceeds one, some degree of selection is inevitable in mutation-accumulation experiments. Also, given that several mutations accumulated in each lineage, the observed fitness values were dependent on genetic interactions. In RNA viruses, mutations tend to have weaker effects as they accumulate (antagonistic epistasis) and the same might hold for ssDNA viruses and small genomes in general [33], probably leading to an underestimate of the mean selection strength in mutation-accumulation experiments.

The fitness effects of random mutations are relevant to evolution in many ways. For instance, they determine the fraction of nucleotide sites that evolves neutrally and thus the rate at which populations diverge through random genetic drift. Further, neutral and deleterious mutations can also determine the rate of adaptive evolution indirectly. The observation that mutations tend to be highly deleterious indicates that there are no efficient buffering mechanisms, which might result in large phenotypic variation and strong selection for beneficial mutations too [34]–[36]. Consistent with this view, the fixation of big-benefit mutations has been reported in several phages [37]–[39] and low mutational robustness has been associated with faster adaptation in VSV [40]. Also, everything else being equal, greater effects of deleterious mutations might result in faster adaptation because strongly deleterious mutations are removed more efficiently from populations, favoring the spread of beneficial ones [41]. The connection between mutational robustness and evolvability is controversial, however. By reducing phenotypic variation, robustness should facilitate the accumulation of genetic variation and may foster evolutionary innovation upon changes in the environment or the genetic background over the long-term [4], [36], [42]–[44], a prediction that has also been supported experimentally [42],[45].

The fitness effects of random mutations are not only relevant to neutral evolution and adaptation, but obviously, also to processes of fitness decline such as Muller's ratchet [46] or lethal mutagenesis [47]. Viruses experience frequent transmission bottlenecks during which random mutations may fix in the population. The consequences of this process for viral fitness are highly dependent on how deleterious are these mutations on average [46]. A substantial proportion of the variation observed in natural populations of RNA viruses comprises transient deleterious mutations, but most fail to reach fixation [48], possibly due to their strongly negative impact on fitness. Mutational fitness effects are also important for understanding viral extinction through mutagenesis [47] and the clinical use of mutagens as ribavirin to treat viral infections makes this subject of practical importance.

A conceptual dichotomy between DNA and RNA viruses has been established based on the observation that the latter evolve faster [49]. However, it has been shown more recently that some ssDNA viruses, including parvoviruses [50], anelloviruses [51] and geminiviruses [52] can match the evolutionary rates of RNA viruses and rapidly adapt to new hosts (although this has not been tested experimentally yet). Despite mutating faster than double-stranded DNA viruses or cellular organisms, ssDNA viruses are less error-prone than ssRNA viruses [19]–[21]. Therefore, the rate at which spontaneous mutations occur may not fully explain why ssRNA and ssDNA viruses evolve at similar rates. Additional factors as, for instance, the fitness effects of mutations, should be considered.

## Materials and Methods

### Bacteriophages and cells

Bacteriophages ΦX174, G4, F1, Qβ, SP, and MS2 and the *E. coli* C strain IJ1862 [29] were obtained from Prof. James J. Bull (University of Texas). The Qβ infectious clone pBRT7Qβ was provided by Dr. René C. Olsthoorn (Leiden University). General biology of the six phages can be found elsewhere [31].

### Preliminary adaptation

Each phage was serially passaged at high population sizes in IJ1862 cells. For each transfer, ∼10^5^ particle forming units (pfu) were inoculated into 0.5 mL LB medium containing IJ1862 cells at their exponential growth phase. The appropriate cell density varied depending on the virus lytic activity and growth rate (OD_600_ = 0.7 for ΦX174 and G4, 0.15 for F1, Qβ, and MS2, and 0.05 for SP). Infected cultures were incubated in agitation (650 rpm) at 37°C in a Thermomixer 24-tube shaker (Eppendorf) and harvested during the late exponential growth phase of the virus (∼10^9^ pfu/mL). Cells were removed by centrifugation and supernatants were aliquoted, stored at −70°C, and titrated using LB medium solidified with soft agar (0.7%). Initial and final titers were used to calculate growth rates and to adjust sampling times for the next passage accordingly. We performed 60–80 passages for each phage. In all cases, no significant changes in growth rate were observed during the last 30 passages, indicating that phages had reached a quasi-maximal fitness under these experimental conditions.

### Mutation-accumulation experiment

Three independent mutation-accumulation lines were seeded for each phage by picking random plaques from the high-fitness populations. Each plaque was resuspended in 50 µL LB and stored at −70°C. Lines were propagated plaque-to-plaque as previously described [53] and after each passage, phages were mutagenized with nitrous acid. To do so, four volumes of 0.3 M acetate buffer pH 4.3 were mixed with one volume of 5 M sodium nitrite and 50 µL of this solution were immediately added to 4 µL of phage-containing supernatant. The exposure time was adjusted to obtain a maximal titer loss. The mutagenesis reactions were quenched by adding 200 µL of 1 M acetate buffer pH 4.3 containing 0.1 mg/mL BSA and 100 µL of this final mix were plated without dilution. After incubation at 37°C, a single lysis plaque was picked, resuspended in 50 µL, aliquoted, stored at −70°C, titrated, and used for the next round of chemical mutagenesis.

### Site-directed mutagenesis

The High Pure Plasmid Isolation kit (Roche) was used to purify ΦX174 DNA and the pBRT7β plasmid from partially lysed *E. coli* IJ1862 cells and from an overnight culture of *E. coli* K12 previously transformed with this cDNA, respectively. For PCR-based mutagenesis, full-length PCR amplicons were obtained from 500 pg of template using Phusion high-fidelity DNA polymerase (New England Biolabs, error rate provided by the manufacturer: 4.4×10^−7^ per base per replication round) and a pair of adjacent, divergent, 5′ phosphorylated primers, one of which carried the desired nucleotide substitution. PCR products were circularized using the Quick T4 ligase (New England Biolabs) and *E. coli* IJ1862 competent cells were transfected by the heat-shock method (42 C, 30 s) in the presence of CaCl_2_ 100 mM. The transfected cells were immediately plated onto LB plates using soft agar and individual plaques were picked after 5-9 hours of incubation at 37°C, resuspended in LB, and stored at −70°C. To verify that the desired mutation had been introduced and that no additional changes were present in the region flanking the target site, (RT)-PCR was performed directly from the resuspended plaque. Moloney murine leukemia virus reverse transcriptase (Fermentas) was used for cDNA synthesis and Phusion DNA polymerase for PCR. The products were column-purified and sequenced using sequence-specific primers.

In cases where transfection yielded no plaques or phages recovered from plaques had not incorporated the mutation, the entire protocol was repeated and, after three consecutive failures, the mutation was classified as a candidate lethal. In all of these cases, the number of plaques obtained from transfection assays was abnormally low, suggesting that the mutation was lethal for the virus and that the few observed plaques came from the template wild-type DNA. To confirm lethality, we first sequenced the region of the PCR-based mutagenesis product flanking the target site to check that the mutation was present and that no additional changes had appeared in this region. Then, we designed new primers that did not carry the nucleotide substitution but were otherwise identical to those used for the mutagenesis reaction, and performed the PCR, circularization and transfection steps exactly as above. Lethality was assessed based on the comparison between the numbers of plaques recovered from mutagenesis versus control reactions (Figure S1).

Similar control assays were carried out to estimate the contribution of non-desired mutations and other sources of error to the inferred distribution of mutational fitness effects. For each phage, we transfected three of the above control PCR-based mutagenesis products and picked 11–12 random plaques from each, yielding 36 and 33 plaques in total for ΦX174 and Qβ, respectively. The relative fitness of each clone was determined as above and we obtained the mean and variance for each phage. These values were subtracted from the means and variances obtained for the real mutants. This allowed us to account for experimental error and, in particular, to control for any potential mutations present in the DNA templates, arising during PCR amplification, or during plaque growth.

### Fitness assays

To measure growth rates, ∼10^4^ pfu were inoculated into 0.5 mL of LB medium containing IJ1862 cells at their exponential growth phase. Infected cultures were incubated in agitation (650 rpm) at 37°C in a Thermomixer 24-tube shaker and harvested when the wild-type reached an estimated titer of 10^8^ pfu/mL. Cells were removed by centrifugation and the supernatants were aliquoted, stored at −70°C and titrated. The growth rate (*r*) was calculated as the increase in log-titer per hour. Relative fitness was obtained as *W* = *r_i_*/*r_0_*, where *i* and *0* refer to the mutant and wild-type, respectively (notice that this fitness measure is in log-scale), and the fitness effect (selection coefficient) was calculated as *s* = *W*−1. In all cases, mutant and wild-type samples were assayed in the same experimental block, and experimental blocks were done in triplicate.

### Sequencing

Sequences were obtained using the Sanger method (Applied Biosystems). In general, we sequenced column-purified plaque-(RT)-PCRs directly. In cases where this yielded low-quality readings, we cloned the PCR fragments using the Zero Blunt TOPO PCR Cloning kit (Invitrogen) and sequenced the inserts with vector-based and internal primers, discarding mutations that were not present in at least 3/5 clones.

### Statistical analyses

For the mutation-accumulation experiment, we used a univariate linear model with two factors: Genetic material (G, main fixed factor) and Phage species (S, random factor nested within G), whereas the line was the experimental replicate. The variables analyzed were the number of passages, the number of mutations fixed, and the expected fitness effect per mutation. For each variable *v*, the model can be written as *ν_ijk_* = *μ*+*G_i_*+*S_ij_*+*l_ijk_*, where *μ* is the grand mean and *l* is the line (error term).

To test for differences in average fitness between the ΦX174 and Qβ mutant collections, we used a univariate linear model with three factors: Phage species (S, main fixed factor), Assay type (A, site-directed mutagenesis or control, fixed factor nested within S), and Plaque (P, each of the mutant or control plaques, random factor nested within A). Hence, each experimental determination can be expressed as *s_ijkl_* = *μ*+*S_i_*+*A_ij_*+*P_ijk_*+*ε_ijkl_*, where *μ* is the grand mean, 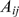 is positive for mutagenesis assays and negative for control assays, and *ε* is the error term.

Non-linear regressions were performed to estimate 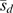 and *V*(*s_d_*) using the Levenberg-Marquardt algorithm implemented in SPSS v12.

## Supporting Information

Figure S1 Confirmation of lethal mutations. Site-directed mutagenesis reactions and control reactions (using non-mutagenic but otherwise identical primers) were loaded in 0.8% agarose gels and stained with ethidium bromide. For each candidate mutation, the left and right lanes contain the mutagenesis and control reactions, respectively. Bands of the expected size (5.4 kb for ΦX174 and ca. 7.0 kb for Qβ insert plus the vector) were obtained in all cases (plus additional, non-specific, lower molecular weight bands which did not interfere with transfections). The number of plaques obtained after transfection of *E. coli* cells with these PCR producuts are shown below the brackets. (A) For ΦX174, all mutations except A402T were confirmed as lethal. (B) For Qβ, all mutations except G780U, G1110A, and G2587U were confirmed.
(0.43 MB PDF)

Table S1 Genetic changes found in ΦX174, G4, F1, Qβ, SP and MS2 mutation accumulation lines (three lines per phage). The final passage number, the relative fitness and its standard error (SEM), the nucleotide substitutions found (genomic position and substitution), the genes where they map, and the associated amino acid change (amino acid number within gene) are shown. Notice that some mutations fall at overlapping genes (the respective amino acid substitutions are indicated in these cases). Genomic positions in ΦX174 are numbered following the convention that nucleotide 1 corresponds to the Pst I cleavage site. Sequences covered nearly the whole genome (>98%) in all cases except for line SP.1 in which we failed to sequence genomic regions 1–167, 653–823, 2277–2633, and 2985–3186, and line MS2.3 in which we failed to sequence region 3048–3569.
(0.03 MB PDF)

Table S2 ΦX174 point mutants obtained by site-directed mutagenesis. For each mutant, the genomic position of the nucleotide substitution (relative to the Pst I cleavage site), the mutated gene, the amino acid change, and the relative fitness effect ± SEM are shown. Assays were done in triplicate and fitness values were corrected to account for the presence of additional mutations and other sources of experimental error (see Materials and Methods). Notice that some mutations fall within regions at overlapping genes (the respective amino acid substitutions are indicated in these cases).
(0.02 MB PDF)

Table S3 Qβ point mutants obtained by site-directed mutagenesis. For each mutant, the genomic position of the nucleotide substitution, the mutated gene, the amino acid change, and the relative fitness effect ± SEM are shown. Assays were done in triplicate and fitness values were corrected to account for the presence of additional mutations and other sources of experimental error (see Materials and Methods). Notice that some mutations fall within regions with overlapping genes (the respective amino acid substitutions are indicated in these cases).
(0.02 MB PDF)
