# Supplementary material for: The Fitness Effects of Random Mutations in Single-Stranded DNA and RNA Bacteriophages
Source: PLoS Genet. 2009 Nov 26;5(11):e1000742. doi: 10.1371/journal.pgen.1000742 (PMC2776273; doi:10.1371/journal.pgen.1000742)
Supplement: Figure S1 — Confirmation of lethal mutations. Site-directed mutagenesis reactions and control reactions (using non-mutagenic but otherwise identical primers) were loaded in 0.8% agarose gels and stained with ethidium bromide. For each candidate mutation, the left and right lanes contain the mutagenesis and control reactions, respectively. Bands of the expected size (5.4 kb for ΦX174 and ca. 7.0 kb for Qβ insert plus the vector) were obtained in all cases (plus additional, non-specific, lower molecular weight bands which did not interfere with transfections). The number of plaques obtained after transfection of E. coli cells with these PCR producuts are shown below the brackets. (A) For ΦX174, all mutations except A402T were confirmed as lethal. (B) For Qβ, all mutations except G780U, G1110A, and G2587U were confirmed. (0.43 MB PDF) [file pgen.1000742.s001.pdf]

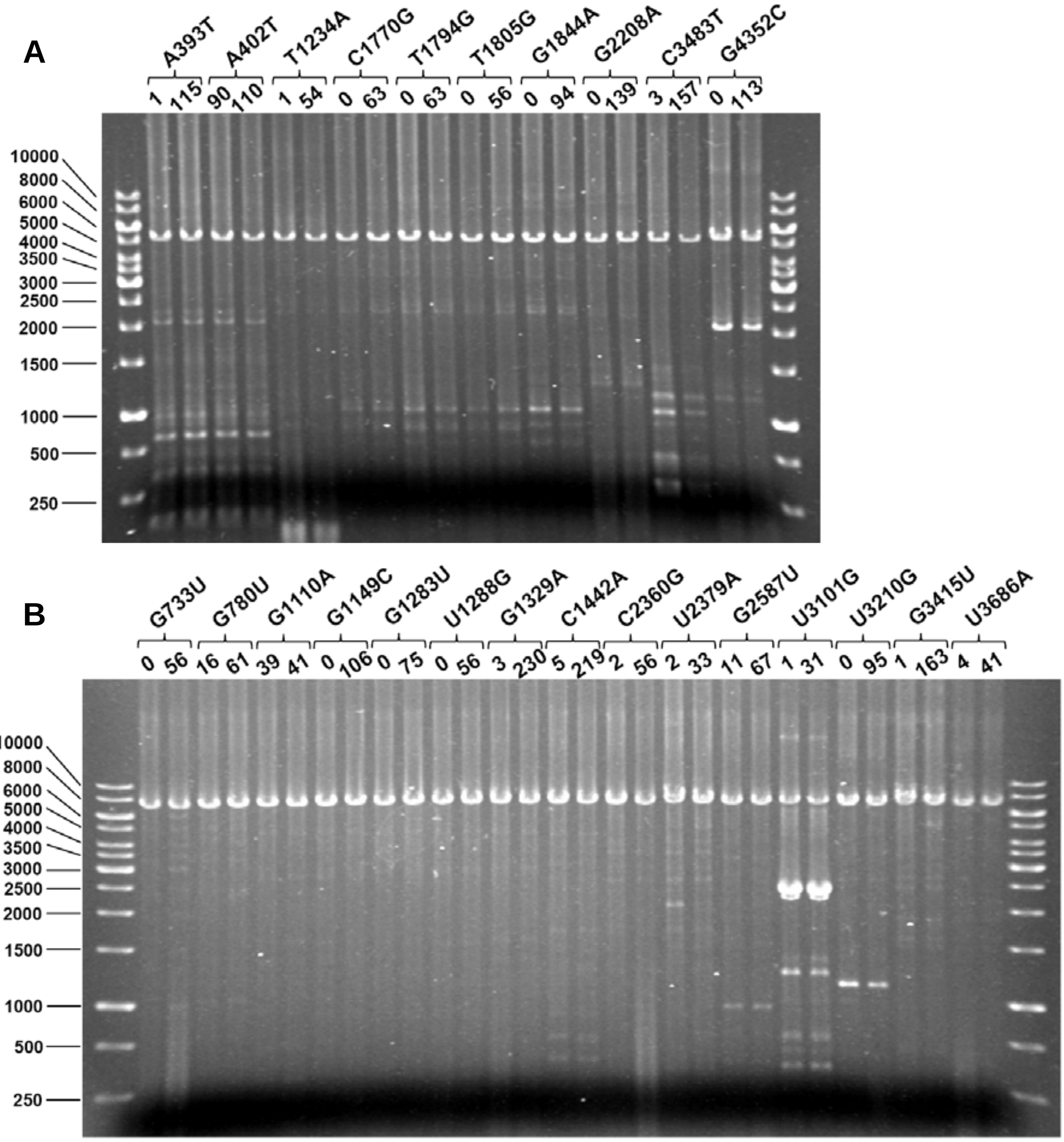


**Fig. S1**. Confirmation of lethal mutations. Site-directed mutagenesis reactions and control reactions (using non-mutagenic but otherwise identical primers) were loaded in 0.8% agarose gels and stained with ethidium bromide. For each candidate mutation, the left and right lanes contain the mutagenesis and control reactions, respectively. Bands of the expected size (5.4 kb for ΦX174 and ca. 7.0 kb for Qβ insert plus the vector) were obtained in all cases (plus additional, non-specific, lower molecular weight bands which did not interfere with transfections). The number of plaques obtained after transfection of *E. coli* cells with these PCR producuts are shown below the brackets. A) For ΦX174, all mutations except A402T were confirmed as lethal. B) For Qβ, all mutations except G780U, G1110A, and G2587U were confirmed.
