# Supplementary material for: The Fitness Effects of Random Mutations in Single-Stranded DNA and RNA Bacteriophages
Source: PLoS Genet. 2009 Nov 26;5(11):e1000742. doi: 10.1371/journal.pgen.1000742 (PMC2776273; doi:10.1371/journal.pgen.1000742)
Supplement: Table S1 — Genetic changes found in ΦX174, G4, F1, Qβ, SP and MS2 mutation accumulation lines (three lines per phage). The final passage number, the relative fitness and its standard error (SEM), the nucleotide substitutions found (genomic position and substitution), the genes where they map, and the associated amino acid change (amino acid number within gene) are shown. Notice that some mutations fall at overlapping genes (the respective amino acid substitutions are indicated in these cases). Genomic positions in ΦX174 are numbered following the convention that nucleotide 1 corresponds to the Pst I cleavage site. Sequences covered nearly the whole genome (>98%) in all cases except for line SP.1 in which we failed to sequence genomic regions 1–167, 653–823, 2277–2633, and 2985–3186, and line MS2.3 in which we failed to sequence region 3048–3569. (0.03 MB PDF) [file pgen.1000742.s002.pdf]

Table S1. Genetic changes found in ΦX174, G4, F1, Qβ, SP and MS2 mutation accumulation lines (three lines per phage). The final passage number, the relative fitness and its standard error (SEM), the nucleotide substitutions found (genomic position and substitution), the genes where they map and the associated amino acid change (amino acid number within gene) are shown. Notice that some mutations fall at overlapping genes (the respective amino acid substitutions are indicated in these cases). Genomic positions in ΦX174 are numbered following the convention that nucleotide 1 corresponds to the Pst I cleavage site. Sequences covered nearly the whole genome (>98%) in all cases except for line SP.1 in which we failed to sequence genomic regions 1-167, 653-823, 2277-2633 and 2985-3186, and line MS2.3 in which we failed to sequence region 3048-3569.

Phage	Lineage	Passage	Relative fitness	Nucleotide substitution	Gene	Amino acid substitution
ΦX 174	1	59	0.228 ± 0.025	G223A	C	Ala31Thr
				T419C	D	None
				A1377G	F	Gln126Arg
				G1417A	F	None
				C2312T	Intergenic	None
				A3253G	H	Lys108Arg
				T3967C	Intergenic	None
				G4166A	A	Met62Ile
				C4329T	A	Arg117Cys
				G4686A	A/A*	Gly236Ser/Gly64Ser
				G5067A	A/A*	Ala363Thr/Ala191Thr
				G5153A	A/A*/B	None/none /Asp27Asn
				G5357T	A/A*/B	None/none /Asp95Tyr
	2	56	0.240 ± 0.024	T121C	A/A*/K	None/none/Leu24Pro
				A424G	D	Gln12Arg
				G774A	D/E	Ala129Thr/none
				G972A	Intergenic	None
				G1258A	F	Met86Ile
				A2361G	Intergenic	None
				G2493A	G	None
				T2676C	G	None
				G3359A	H	Met143Ile
				G3470A	H	None
				G4592A	A/A*	None/none
				A4657G	A/A*	Asp226Gly/Asp54Gly
				A5090G	A/A*/B	None/none/Lys6EGlu
	3	66	0.238 ± 0.031	G50A	A/A*	Asp486Asn/Asp314Asn
				G210A	K/C	Val54Ile/none
				G394A	D	Ser2Asn
				A445G	D	Lys19Arg
				G731A	D/E	None/Arg55Gln
				A827G	D/E	None/Tyr87Cys
				T1000C	Intergenic	None
				A1091G	F	Ile31Val
				G1268A	F	Val90Ile
				G1537A	F	None
				A2224G	F	None
				A2306G	Intergenic	None
				G2984A	H	Met18Ile
				G3333A	H	Gly135Ser

				A3515G	H	None
				G3611A	H	None
				G3704A	H	None
				T3790C	H	Val287Ala
				T4165C	A	Met62Thr
				G4994A	A/A*	Met338Ile/Met166Ile
				A5040G	A/A*	Met354Val/Met182Val
				G5059A	A/A*	Gly360Asp/Gly188Asp
G4	1	26	0.115 ± 0.030	A226G	A	None
				A610C	A	None
				G691A	A	None
				G1667A	A/A*/K	Gly537Arg/Gly324Arg/none
				A1683G	A/A*/K	Asn542Ser/Asn329Ser/Thr16Ala
				A2159G	D/E	Thr62Ala/none
				T2633C	F	Ser12Pro
				A3305C	F	Ile236Leu
				G3463A	F	None
				A3526G	F	None
				C4571G	H	Ala3Gly
				C4580T	H	Ala6Val
				A4688G	H	Asn42Ser
				G4762T	H	Ala67Ser
				T4809C	H	None
				A4828G	H	Asn89Asp
	2	57	0.457 ± 0.019	G700A	A/A*	Met214Ile/Met1Ile
				G1267A	A/A*	None/none
				A1533G	A/A*/B	Asn492Ser/Asn279Ser/none
				A1565G	A/A*/B	Thr503Ala/Thr290Ala/Asn97Ser
				A1823G	C	Glu35Gly
				A2036G	D	Ile21Val
				G2306A	D/E	Val111Ile/none
				A2437G	E	Asp95Gly
				G5148C	H	None
				G5314A	H	Gly251Ser
	3	19	0.368± 0.018	C285T	A	Ala76Val
				A315G	A	Lys86Arg
				G991A	A/A*	Met311Ile/Met98Ile
				A1449G	A/A*/B	Lys464Arg/Lys251Arg/none
				A3833G	F	Asn412Asp
				C4746T	H	None
F1	1	53	0.261 ± 0.055	A58G	pII	Asn154Asp
				G280A	pII	Val228Ile
				G1037A	pV	None
				G1050A	pV	Val70Ile
				A1727T	pIII	Asn50Ile
				A1937G	pIII	Tyr120Cys
				G2428A	pIII	Ala284Thr
				C2468T	pIII	Ala297Val
				T2478C	pIII	None
				G2567A	pIII	Gly330Asp
				G2594A	pIII	Gly339Asp
				T2812C	pIII	Phe412Leu
				G2820A	pIII	None
				G2835A	pIII	None
				G2862A	pVI	Val3Ile
				A3348G	pI	None

			T3522C	pI	None
			G4044A	pI	None
			A4502G	pIV	Asn95Asp
			G4544A	pIV	Asp109Asn
			G4763A	pIV	Asp182Asn
			G4853A	pIV	Ala212Thr
			A5833G	Intergenic	None
			G5875A	Intergenic	None
			G5978A	Intergenic	None
			G5999A	Intergenic	None
			G6069A	pII	Gly22Ser
			G6121A	pII	Gly39Asp
			G6159A	pII	Asp52Asn
2	58	0.580 ± 0.052	G388A	pII	Asp264Asn
			G537A	pII/pX	None/none
			G748A	pII/pX	Val384Ile/Val85Ile
			G1309A	pVIII	None
			G1360A	pVIII	None
			G1415A	pVIII	Ala39Thr
			A1593G	pIII	None
			G1609A	pIII	Val11Ile
			A1667G	pIII	His30Arg
			G1837A	pIII	Gly87Ser
			G1866A	pIII	None
			C1887T	pIII	None
			G1912A	pIII	Asp112Asn
			T2118C	pIII	None
			T2239C	pIII	Tyr221His
			G2353A	pIII	Gly259Ser
			A2425G	pIII	Asn283Asp
			G2449A	pIII	Glu291Lys
			T2983C	pVI	Phe43Ser
			G3089A	pVI	None
			T3261C	pI	None
			G4544A	pIV	Asp109Asn
			G4838A	pIV	Val207Ile
			G5382A	pIV	Gly388Asp
			G5616C	Intergenic	None
			C5684T	Intergenic	None
			G5952A	Intergenic	None
			G6084A	pII	Ala27Thr
3	26	0.185 ± 0.036	G300A	pII	None
			C315T	pII	None
			G1774A	pIII	Gly66Ser
			G1859A	pIII	Gly94Asp
			G1883A	pIII	Gly102Asp
			T2466C	pIII	None
			A2491G	pIII	Lys305Glu
			G2566A	pIII	Gly330Ser
			A2760G	pIII	None
			G4745A	pIV	Ala176Thr
			G4984A	pIV	None
			A5085G	pIV	Gln289Arg
			C5825T	Intergenic	None
			G5969A	Intergenic	None
			G6143A	pII	None
			G6194A	pII	None

				A6214G	pII	His70Arg
				G6228A	pII	Ala75Thr
				G6281A	pII	None
				G6350A	pII	None
Qβ	1	19	0.257 ± 0.045	A424G	Maturation	None
				A713G	Maturation	Arg219Gly
				A1063G	Maturation	None
				A1312G	Maturation	None
				A1452G	Coat/read-through	None/none
				A1567G	Coat/read-through	Thr76Ala/Thr76Ala
				U1626C	Coat/read-through	None/none
				C1692U	Coat/read-through	None/none
				U2001C	Read Through	None
				C2244U	Read Through	None
				G2350A	replicase	Val1Met
				A2427G	Replicase	None
				C2526U	Replicase	None
				C2560U	Replicase	Leu71Phe
				A2763G	Replicase	None
				U2967C	Replicase	None
				U3101G	Replicase	Leu251Arg
				U3369C	Replicase	None
				A3601G	Replicase	Ser418Gly
				A3651G	Replicase	None
				C3657U	Replicase	None
				A4135G	Intergenic	None
	2	7	0.228 ± 0.030	C285U	Maturation	Ser76Leu
				A357G	Maturation	Asp100Gly
				U469C	Maturation	None
				A550G	Maturation	None
				U994C	Maturation	None
				A1051G	Maturation	Ile331Met
				A1136G	Maturation	Ile360Val
				C1293U	Maturation	Ser412Phe
				U1662C	Coat/read-through	None/none
				G2262A	Read-through	None
				C2307U	Read-through	None
				A2439C	Replicase	None
				A2931G	Replicase	None
				A3848G	Replicase	Asp500Gly
	3	17	0.400 ± 0.024	C262U	Maturation	None
				C407U	Maturation	None
				C624U	Maturation	Ser189Phe
				A819G	Maturation	Tyr2154Cys
				U823C	Maturation	None
				C826A	Maturation	None
				A903G	Maturation	Glu282Gly
				A1141G	Maturation	None
				A1383G	Coat/read-through	None/none
				A1885G	Read-through	Thr18Ala2
				A1913G	Read-through	Asn191Ser
				A1972G	Read-through	Thr211Ala
				C2382U	Replicase	None
				C2411U	Replicase	Thr21Ile
				A2488G	Replicase	Asn47Asp
				A2555G	Replicase	Asn69Ser
				A3154G	Replicase	Asn269Asp

				A3796G	Replicase	Lys483Glu
				A3841G	Replicase	Thr498Ala
				A3977G	Replicase	Gln543Arg
SP	1	11	0.109 ± 0.049	A208G	Maturation	Asn52Asp
				U236C	Maturation	Val61Ala
				U294C	Maturation	None
				G362A	Maturation	Gly103Asp
				G537C	Maturation	None
				G1099A	Maturation	None
				C1290U	Maturation	None
				C1548U	Coat/read-through	Ala41Val/Ala41Val
				C1699U	Coat/read-through	None/none
				G1856A	Read-through	Gly144Arg
				G2141C	Read-through	Asp239His
				C2752U	Replicase	Thr104Met
				C2931U	Replicase	His164Tyr
				G3381A	Replicase	Val314Ile
				C4242U	Intergenic	None
	2	4	0.122 ± 0.021	A648G	Maturation	None
				G1007A	Maturation	Arg103His
				A1321G	Maturation	Ile423Val
				C1908G	Read-through	Pro161Arg
				U1997C	Read-through	Tyr191His
				A2523G	Replicase	Met28Val
				C2707U	Replicase	Ala89Val
				A2779G	Replicase	Tyr113Cys
				C3149U	Replicase	None
				A3456G	Replicase	Ile339Val
				A3796G	Replicase	Lys452Arg
	3	6	0.383 ± 0.024	A516G	Maturation	None
				A1332G	Maturation	None
				A1457G	Coat/read-through	Lys11Glu/Lys11Glu
				A1486G	Coat/read-through	None/none
				U1627G	Coat/read-through	Ile67Met/Ile67Met
				C1639G	Coat/read-through	Asn71Lys/Asn71Lys
				C1653A	Coat/read-through	Thr76Lys/Thr76Lys
				C1674U	Coat/read-through	Ser83Phe/Ser83Phe
				C1682A	Coat/read-through	None/none
				C1981G	Read-through	Asp185Glu
				A2003G	Read-through	Arg193Gly
				G2012A	Read-through	Glu196Lys
				A2014G	Read-through	Glu196Lys
				U2016G	Read-through	Val197Gly
				A2392G	Read-through	None
				U2419A	Read-through	None
				A2499G	Replicase	Ile20Val
				A3394G	Replicase	Glu318Gly
				C3581U	Replicase	None
MS2	1	8	0.473 ± 0.101	A333G	Assembly	None
				A845G	Assembly	Lys239Arg
				C894U	Assembly	None
				C1125U	Assembly	None
				A1126G	Assembly	Ile333Val
				A1185G	Assembly	None
				A2036G	Replicase	None
				A2363G	Replicase	None

			A2466G	Replicase	Asn236Asp
			C2534U	Replicase	None
			A3037G	Replicase	Tyr426Cys
			A3226G	Replicase	Lys489Arg
			A3303G	Replicase	Lys515Glu
2	7	0.215 ± 0.027	A436G	Assembly	Ser103Gly
			A541G	Assembly	Asn138Asp
			A614G	Assembly	Lys162Arg
			C961A	Assembly	Leu278Arg
			U962G	Assembly	Leu278Arg
			A963G	Assembly	Leu278Arg
			G964U	Assembly	Gly279Cys
			C1104U	Assembly	None
			C1637U	Coat	None
			A1860G	Lysis/Replicase	None/Ser34Gly
			C1979U	Replicase	None
			A2641G	Replicase	Asp294Gly
			A2732U	Replicase	Lys324Asn
			U3138G	Replicase	Ser460Ala
			A3143G	Replicase	None
			A3519G	Intergenic	None
3	7	0.250 ± 0.012	C354U	Assembly	None
			A361G	Assembly	Ser78Gly
			A884G	Assembly	Gln252Arg
			G937C	Assembly	Ala270Pro
			U944C	Assembly	Leu272Ser
			G946C	Assembly	Ala273Pro
			G1119U	Assembly	Glu330ASp
			A1166G	Assembly	Gln346Arg
			A1535G	Coat	None
			U1632C	Coat	Ser100Pro
			C1712U	Coat/Lysis	None/Thr12Ile
			A1920G	Replicase	Tyr54Ala
			C2009U	Replicase	None
			A2142G	Replicase	Tyr128Ala
			A2249U	Replicase	None
			A2447G	Replicase	None
