# Supplementary material for: The Fitness Effects of Random Mutations in Single-Stranded DNA and RNA Bacteriophages
Source: PLoS Genet. 2009 Nov 26;5(11):e1000742. doi: 10.1371/journal.pgen.1000742 (PMC2776273; doi:10.1371/journal.pgen.1000742)
Supplement: Table S2 — ΦX174 point mutants obtained by site-directed mutagenesis. For each mutant, the genomic position of the nucleotide substitution (relative to the Pst I cleavage site), the mutated gene, the amino acid change, and the relative fitness effect ± SEM are shown. Assays were done in triplicate and fitness values were corrected to account for the presence of additional mutations and other sources of experimental error (see Materials and Methods). Notice that some mutations fall within regions at overlapping genes (the respective amino acid substitutions are indicated in these cases). (0.02 MB PDF) [file pgen.1000742.s003.pdf]

Table S2. ΦX174 point mutants obtained by site-directed mutagenesis. For each mutant, the genomic position of the nucleotide substitution (relative to the Pst I cleavage site), the mutated gene, the amino acid change and the relative fitness effect ± SEM are shown. Assays were done in triplicate and fitness values were corrected to account for the presence of additional mutations and other sources of experimental error (see Methods). Notice that some mutations fall within regions at overlapping genes (the respective amino acid substitutions are indicated in these cases).

Mutation	Gene	Amino acid substitution	Relative fitness effect
C357A	C	None	-0.001 ± 0.009
A393T	C/D	STOP87Cys/Ser2Cys	-1 (lethal)
A510T	D	Lys41STOP	-0.151 ± 0.026
T825A	D/E	Leu146Ile/Asn86Lys	-0.348 ± 0.105
A827T	D/E	Leu146Ile/Tyr87Phe	-0.105 ± 0.071
G915C	J	Gly23Ala	-0.063 ± 0.09
T1006G	F	None	-0.191 ± 0.072
C1023A	F	Ala8Asp	-0.314 ± 0.036
C1174T	F	None	-0.122 ± 0.02
C1217T	F	Pro73Ser	-0.306 ± 0.057
T1234A	F	Tyr78STOP	-1 (lethal)
G1238C	F	Glu80Gln	-0.052 ± 0.032
A1251C	F	Lys84Thr	0.035 ± 0.026
A1295C	F	Asn99Thr	-0.041 ± 0.030
T1432A	F	None	0.068 ± 0.013
T1516C	F	None	0.015 ± 0.026
T1663G	F	None	0.023 ± 0.057
C1770G	F	Ser257Try	-1 (lethal)
T1794G	F	Val265Gly	-1 (lethal)
T1805G	F	Tyr269Asp	-1 (lethal)
G1844A	F	Gly282Ser	-1 (lethal)
T1870G	F	None	-0.142 ± 0.148
G2208A	F	Try403STOP	-1 (lethal)
T2350A	Intergenic	None	-0.138 ± 0.029
G2511T	G	None	0.022 ± 0.019
T2562A	G	Phe56Leu	-0.430 ± 0.084
T2630A	G	Ile79Asn	-0.205 ± 0.061
T3233G	H	Asp101Glu	0.061 ± 0.03
G3236A	H	None	0.059 ± 0.043
G3377T	H	Glu149Asp	0.001 ± 0.017
G3422C	H	Glu164Asp	0.020 ± 0.019
C3483T	H	Arg185Cys	-1 (lethal)
T3569C	H	None	0.019 ± 0.035
G3599T	H	Gln223His	0.006 ± 0.024
G3683C	H	Met251Ile	-0.016 ± 0.005
T4201A	A	Val74Asp	-0.518 ± 0.023
G4352C	A	Try124Cys	-1 (lethal)
A4458C	A	Ile160Leu	-0.037 ± 0.023
T4628G	A/A*	Asn216Lys/Asn44Lys	0.061 ± 0.124
T4643G	A/A*	None/none	-0.074 ± 0.034
T4748C	A/A*	None/none	-0.074 ± 0.004
C4972G	A/A*	Pro331Arg/Pro159Arg	-0.66 ± 0.024
C5192G	A/A*/B	Cys404Try/Cys232Try/Leu40Val	-0.889 ± 0.033
A5287G	A/A*/B	Lys436Arg/Lys264Arg/ None	0.006 ± 0.001
T5344G	A/A*/B	Phe455Cys/ Phe283Cys/Ile90Met	-0.057 ± 0.028
