# Supplementary material for: The Fitness Effects of Random Mutations in Single-Stranded DNA and RNA Bacteriophages
Source: PLoS Genet. 2009 Nov 26;5(11):e1000742. doi: 10.1371/journal.pgen.1000742 (PMC2776273; doi:10.1371/journal.pgen.1000742)
Supplement: Table S3 — Qβ point mutants obtained by site-directed mutagenesis. For each mutant, the genomic position of the nucleotide substitution, the mutated gene, the amino acid change, and the relative fitness effect ± SEM are shown. Assays were done in triplicate and fitness values were corrected to account for the presence of additional mutations and other sources of experimental error (see Materials and Methods). Notice that some mutations fall within regions with overlapping genes (the respective amino acid substitutions are indicated in these cases). (0.02 MB PDF) [file pgen.1000742.s004.pdf]

**Table S3.** Qβ point mutants obtained by site-directed mutagenesis. For each mutant, the genomic position of the nucleotide substitution, the mutated gene, the amino acid change, and the relative fitness effect ± SEM are shown. Assays were done in triplicate and fitness values were corrected to account for the presence of additional mutations and other sources of experimental error (see Methods). Notice that some mutations fall within regions with overlapping genes (the respective amino acid substitutions are indicated in these cases).

| Mutation | Gene | Amino acid substitution | Relative fitness effect |
|---|---|---|---|
| A161C [1] | Maturation | Thr35Pro | -0.007 ± 0.007 |
| A328G | Maturation | None | 0.003 ± 0.029 |
| C665G | Maturation | Leu203Val | -0.440 ± 0.016 |
| G733U | Maturation | Try225Cys | -1 (lethal) |
| U772G | Maturation | Phe238Leu | -0.365 ± 0.078 |
| A891U | Maturation | Tyr278Phe | -0.095 ± 0.056 |
| C939G | Maturation | Pro294Arg | -0.072 ± 0.023 |
| C1096U | Maturation | None | -0.026 ± 0.045 |
| G1149C | Maturation | Arg364Pro | -1 (lethal) |
| U1165A | Maturation | None | -0.039 ± 0.017 |
| G1283U | Maturation | Asp409Tyr | -1 (lethal) |
| U1288G | Maturation | Ser410Arg | -1 (lethal) |
| G1329A | Intergenic | Intergenic | -1 (lethal) |
| C1442A | Read-through/ Coat | Ala34Asp/Ala34Asp | -1 (lethal) |
| U1485A | Read-through/ Coat | None/none | -0.043 ± 0.052 |
| A1525U | Read-through/Coat | Asn62Tyr /Asn62Tyr | 0.035 ± 0.027 |
| U1809C | Read-through | None | -0.027 ± 0.061 |
| C1814U | Read-through | Pro158Leu | -0.014 ± 0.041 |
| G1895U | Read-through | Arg185Leu | -0.059 ± 0.012 |
| A2090U | Read-through | Gln250Leu | -0.230 ± 0.011 |
| C2360G | Replicase | Thr4Arg | -1 (lethal) |
| U2379A | Replicase | None | -1 (lethal) |
| G2787U | Replicase | Glu146Asp | -0.098 ± 0.010 |
| G2793U | Replicase | Met148Ile | -0.093 ± 0.031 |
| C2797A | Replicase | Arg150Ser | -0.081 ± 0.011 |
| U3027C | Replicase | None | 0.025 ± 0.032 |
| U3075C | Replicase | None | 0.000 ± 0.044 |
| A3094G | Replicase | Ile249Val | 0.007 ± 0.003 |
| U3101G | Replicase | Leu251Arg | -1 (lethal) |
| U3210G [2] | Replicase | Cys287Try | -1 (lethal) |
| G3213U | Replicase | Glu288Asp | -0.047 ± 0.038 |
| G3397C | Replicase | Asp350His | -0.260 ± 0.034 |
| G3415U | Replicase | Val356Phe | -1 (lethal) |
| U3452C | Replicase | Val368Ala | -0.329 ± 0.019 |
| U3483G | Replicase | None | 0.016 ± 0.014 |
| G3661U | Replicase | Val438Leu | -0.223 ± 0.02 |
| U3686A | Replicase | Val446Glu | -1 (lethal) |
| G3815C | Replicase | Arg489Pro | 0.032 ± 0.041 |
| G3916C | Replicase | Asp523His | -0.228 ± 0.038 |
| U3951G | Replicase | Asp534Glu | -0.004 ± 0.041 |
| G4066U | Replicase | Ala573Ser | -0.069 ± 0.091 |
| U4082A [1] | Replicase | Phe578Tyr | -0.352 ± 0.019 |

[1] The presence of the mutation could not be confirmed by sequencing.
[2] Despite the mutation was classified as lethal (Fig. S1A), mutant viruses with apparently neutral fitness were successfully recovered from a single plaque. A likely explanation is that the mutation was quasi-lethal and had partially reverted or been compensated by a secondary mutation in the isolated plaque.
